# Microscale thermophoresis suggests a new model of regulation of cardiac myosin function *via* interaction with cardiac myosin-binding protein C

**DOI:** 10.1016/j.jbc.2021.101485

**Published:** 2021-12-14

**Authors:** Saraswathi Ponnam, Thomas Kampourakis

**Affiliations:** Randall Centre for Cell and Molecular Biophysics; and British Heart Foundation Centre of Research Excellence, King's College London, London, United Kingdom

**Keywords:** cardiac muscle regulation, cardiac myosin, myosin-binding protein C, phosphorylation, hypertrophic cardiomyopathy, cMLCK, cardiac isoform of myosin light chain kinase, cMyBP-C, cardiac isoform of myosin-binding protein C, C0C2, cMyBP-C spanning domains C0 to C2, C0C2-3P, Tris-phosphorylated C0C2, ESI, electrospray ionization, HCM, hypertrophic cardiomyopathy, Ig, immunoglobulin, IHM, interacting-heads motif, MST, microscale thermophoresis, myosin S2Δ, myosin subfragment-2, NTD, N-terminal domain, NTF, native thin filament, P/A, proline- and alanine-rich, PKA, protein kinase A, RLC, regulatory light chain, TEV, tobacco etch virus

## Abstract

The cardiac isoform of myosin-binding protein C (cMyBP-C) is a key regulatory protein found in cardiac myofilaments that can control the activation state of both the actin-containing thin and myosin-containing thick filaments. However, in contrast to thin filament–based mechanisms of regulation, the mechanism of myosin-based regulation by cMyBP-C has yet to be defined in detail. To clarify its function in this process, we used microscale thermophoresis to build an extensive interaction map between cMyBP-C and isolated fragments of β-cardiac myosin. We show here that the regulatory N-terminal domains (C0C2) of cMyBP-C interact with both the myosin head (myosin S1) and tail domains (myosin S2) with micromolar affinity *via* phosphorylation-independent and phosphorylation-dependent interactions of domain C1 and the cardiac-specific m-motif, respectively. Moreover, we show that the interaction sites with the highest affinity between cMyBP-C and myosin S1 are localized to its central domains, which bind myosin with submicromolar affinity. We identified two separate interaction regions in the central C2C4 and C5C7 segments that compete for the same binding site on myosin S1, suggesting that cMyBP-C can crosslink the two myosin heads of a single myosin molecule and thereby stabilize it in the folded OFF state. Phosphorylation of the cardiac-specific m-motif by protein kinase A had no effect on the binding of either the N-terminal or the central segments to the myosin head domain, suggesting this might therefore represent a constitutively bound state of myosin associated with cMyBP-C. Based on our results, we propose a new model of regulation of cardiac myosin function by cMyBP-C.

The cardiac contraction–relaxation cycle is driven by the calcium-dependent interaction of the myosin-containing thick and actin-containing thin filaments. The calcium-induced activation of the thin filaments at the beginning of systole allows myosin head domains from the neighboring thick filaments to strongly attach to actin. Fueled by the hydrolysis of ATP, myosin heads perform the working stroke, leading to nanometer-scale displacement of the thin filaments toward the center of the sarcomere or the development of piconewton-scale forces. Conversely, removal of cytoplasmic calcium ions from the sarcomere deactivates the thin filaments, followed by the detachment of myosin heads from actin and the onset of mechanical relaxation (1).

In addition to the canonical calcium-dependent thin filament–based regulatory pathway described previously, recent studies suggested an additional permissive step during cardiac muscle activation, involving a structural transition in the thick filaments associated with the release of myosin heads from a quasi-helical arrangement on the surface of the thick filaments (OFF or super-relaxed state, SRX) into a functional ON or disordered-relaxed (DRX) state (Fig. 1*A*) (2, 3). Although no high-resolution structures of the cardiac myosin OFF state are currently available, biochemical and biophysical studies suggests that myosin heads adopt a folded conformation in which their ATPase activity and actin-binding capability are strongly inhibited (4, 5). This folded conformation is characterized by an asymmetric interaction of the two myosin heads of single molecule, with the so-called “free head” sitting on top of the “blocked head” (4, 6, 7). This OFF conformation is believed to be stabilized *via* myosin head–head and head–tail domain interactions (Fig. 1*B*) and interaction between the heads and tails with thick filament accessory proteins such as titin and myosin-binding protein C (8, 9, 10).

**Figure 1:**
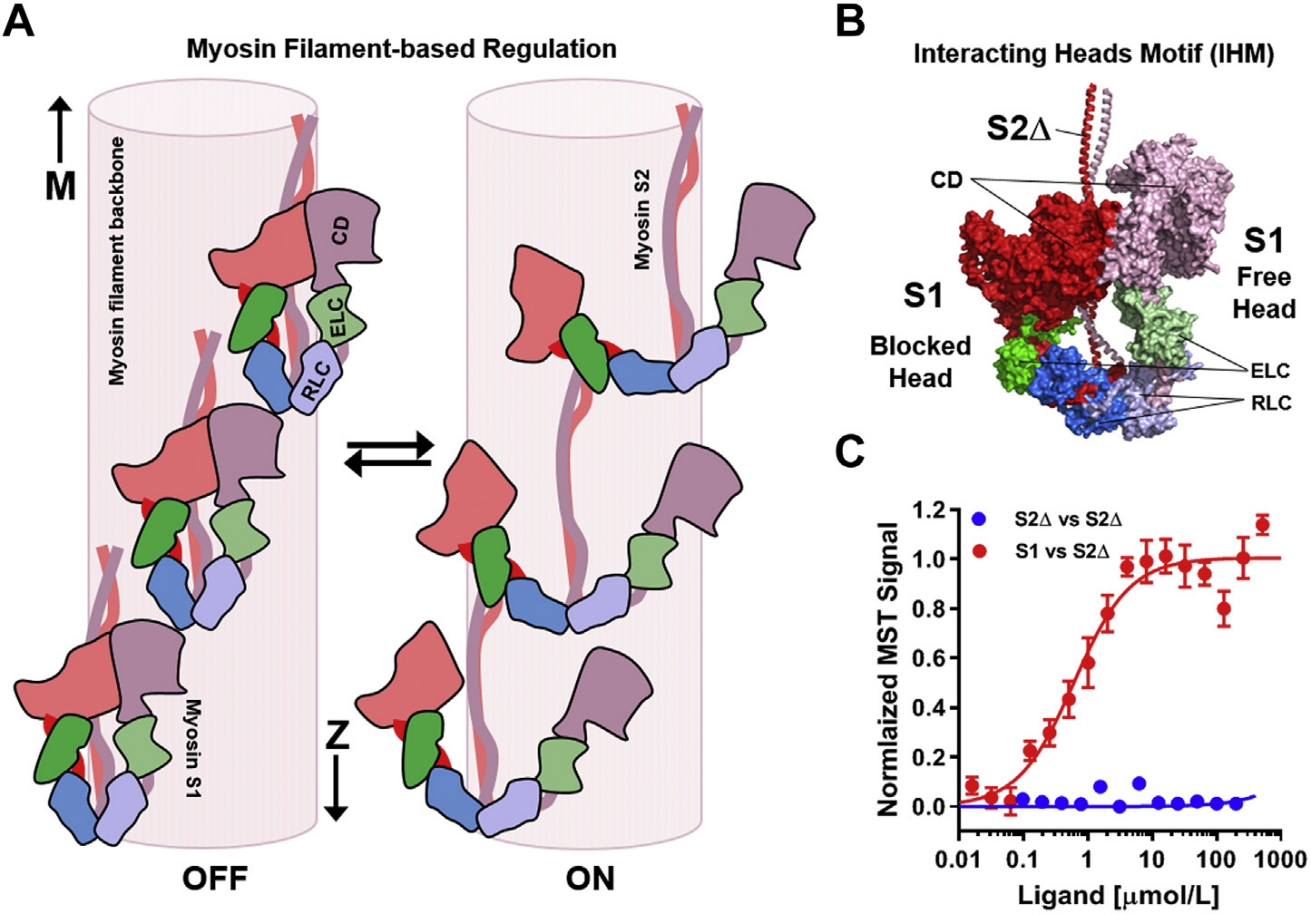
**Myosin filament–based regulation in the heart.***A,* cartoon representation of the myosin filament OFF (*left*) and ON state (*right*). The myosin catalytic domain (CD), essential light chain (ELC), and regulatory light chain (RLC) are shown *red*, *green*, and *blue*, respectively. The positions of M-band and Z-disc are indicated accordingly. *B,* atomic model of cardiac myosin in the interacting heads motif (Protein Data Bank: 5TBY). Blocked head and free head and myosin tails (S2Δ) are labeled accordingly. *C,* normalized MST binding curves for myosin S2Δ titrated against Alexa647-labeled myosin S1 (*red*) or myosin S2Δ (*blue*). Means ± SEM, n = 8. MST, microscale thermophoresis.

The cardiac isoform of myosin-binding protein-C (cMyBP-C) is part of a group of sarcomeric proteins that are frequently found mutated in patients suffering from hypertrophic cardiomyopathy (HCM) associated heart failure, underlining its functional significance in controlling cardiac myofilament function. Moreover, knockout or mutation of cMyBP-C lead to the development of HCM and heart failure in transgenic animal models (11), suggesting that its native structure is required for the normal performance and energy efficiency of the vertebrate heart. Gene knockout of cMyBP-C decreased the calcium sensitivity but accelerated crossbridge kinetics of isolated cardiac muscle (12), indicating that cMyBP-C might have both activating and inhibiting effects on myocardial contractility.

cMyBP-C is composed of interspersed eight immunoglobulin (Ig)-like and three fibronectin-like domains termed C0 through C10. The C-terminal regions spanning domains C8 through C10 anchor cMyBP-C to the so-called C-zone, corresponding to the inner two-thirds of the sarcomeric A-band, *via* interactions with both light meromyosin and titin super-repeats (13, 14). The N-terminal domains (NTDs) C0 and C1 are connected *via* a proline- and alanine-rich (P/A) linker of about 50 to 60 amino acids, followed by the partially unstructured m-motif linking domains C1 and C2, which contains cardiac-specific phosphorylation sites for physiologically and pathophysiologically relevant protein kinases (15). The NTDs of cMyBP-C have been shown to interact with both the actin-containing thin filaments and myosin-containing thick filaments both *in vitro* and *in situ* (16, 17), interactions that are associated with a change in the filament regulatory states. Actin filament binding of NTDs of cMyBP-Cs has been shown to activate the thin filament structure in the absence of Ca^2+^ (16, 18, 19). This increases the calcium sensitivity of the thin filaments and force generation. In contrast, NTD binding to myosin has been generally associated with a stabilization of the myosin head domain OFF state and reduced contractility (8, 16).

In contrast to the NTD and C-terminal domain, the role of the central segment of cMyBP-C has remained largely elusive, although cardiac-specific sequence insertions and the clustering of missense mutations in the central domains suggest a function beyond a simple spacer that holds the NTDs close to actin-binding and myosin-binding sites (20, 21).

Both the inhibitory and activating effects of the interactions of cMyBP-C are believed to be physiologically controlled by its phosphorylation state. For instance, the effects of β-adrenergic stimulation on cardiac contractile function, such as increased crossbridge kinetics and rate of myofilament relaxation (22), are partially mediated by the protein kinase A (PKA)-dependent phosphorylation of cMyBP-C. Phosphorylation of key sites within the cardiac-specific m-motif is thought to activate the thick filament structure *via* release of myosin head domains from the filament core into the functional ON or disordered relaxed state, thereby increasing their ATPase activity and the probability for binding to actin (23, 24). In good agreement with the functional studies, HCM-associated mutations in both β-cardiac myosin and cMyBP-C have been shown to interfere with the myosin head OFF state stabilized by cMyBP-C (8, 10). This traps myosin heads in the ON or DRX state (25), which is expected to increase the contractility and energy demand of the myocardium—both hallmarks of HCM. However, the structural basis of the cMyBP-C-based regulation of cardiac myosin function and its impairment during disease states of the heart has remained largely elusive.

In this study, we examined the interaction of the domains of cMyBP-C with several regions of isolated β-cardiac myosin using a quantitative approach *via* microscale thermophoresis (MST), which allowed us to construct a detailed protein–protein interaction map that includes information on binding parameters. While previous studies solely focused on the phosphorylation-sensitive interaction of the m-motif with myosin S2 (26, 27), we identified the C1/m-motif interface as a phosphorylation-dependent regulatory element that binds close to the myosin head–rod junction.

Moreover, we show that the regulatory interaction sites with the highest affinity between cMyBP-C and the myosin head domains are not solely localized to the phosphorylatable NTD but also in its central segment. This suggests that the central domains of cMyBP-C are not a passive element that keeps the NTDs near their actin-binding and myosin-binding sites but play a dynamic regulatory role to control the conformation and function of the myosin heads. Thus, we propose a new model of contractile regulation by cMyBP-C that explains the functional effects of cMyBP-C phosphorylation by increasing the probability of its NTDs to bind actin and subsequent release myosin heads from the OFF or SRX state.

## Results

### Cardiac myosin heads are tightly bound to their tail domains

Here, we probed the interaction of isolated cardiac myosin heads (myosin S1) with their tail domains using MST. Alexa 647-labeled bovine β-cardiac myosin S1 was titrated against increasing concentrations of recombinant human cardiac myosin tail domain, encompassing the first 126 amino acids of myosin subfragment-2 (myosin S2Δ) (Fig. 1*C*, *red*). Normalized MST data were fitted to a logistic binding curve, resulting in a steady-state dissociation constant *K*_*d*_ of 0.6 ± 0.1 μmol/l (mean ± SEM, n = 8), in good agreement with previously published results using recombinant human β-cardiac myosin head domain (8). As a negative control, we titrated unlabeled myosin S2Δ into Alexa 647-labeled myosin S2Δ, which showed no binding *via* MST (Fig. 1*C*, *blue*).

The local or effective concentrations of both myosin heads and tails in native thick filaments were previously estimated to be in the medium to high micromolar range (8, 17), which is several orders of magnitude higher than the measured *K*_*d*_ value for the head–tail interaction, suggesting that in the absence of other available binding partners one of the two myosin heads of a myosin molecule (*i.e.*, the “blocked head”) is tightly bound to its tail domain. In contrast, the partner head (*i.e.*, the “free head”) is likely less restrained and more mobile.

### NTDs of cMyBP-C bind both myosin head and tail domains with moderate affinity

**Figure 2:**
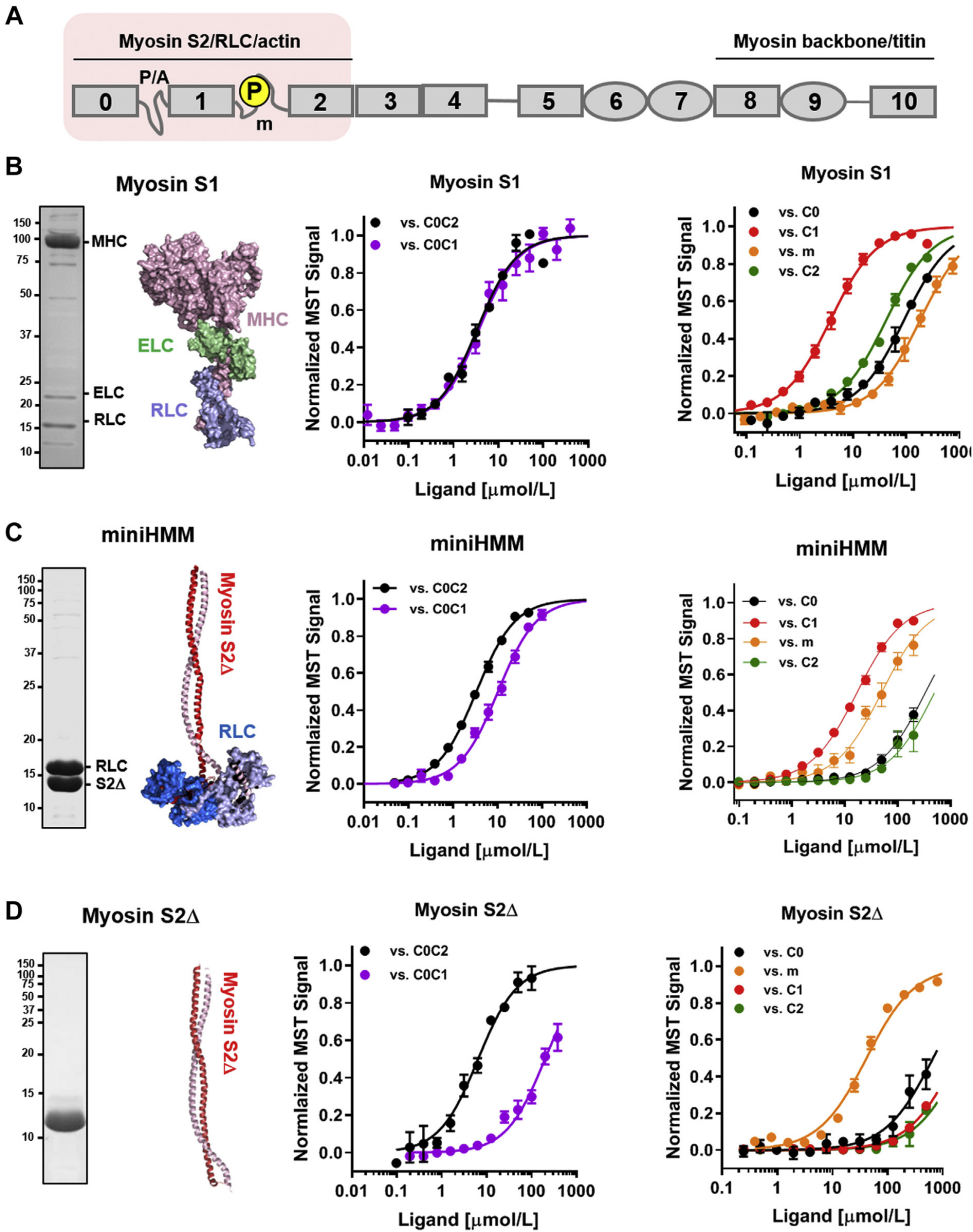
**Interaction of N-terminal domains of cMyBP-C with cardiac myosin.***A,* domain structure and known interaction partners of cMyBP-C. The P/A linker and m-motif (m) are labeled accordingly. *B, left,* SDS-PAGE and structure of isolated myosin S1 with myosin heavy chain (MHC), essential light chain (ELC), and regulatory light chain (RLC) labeled accordingly. *Right,* normalized MST binding curves for different cMyBP-C fragments titrated against myosin S1. *C, left,* SDS-PAGE and structure of miniHMM with myosin tail part (myosin S2Δ) and RLC labeled accordingly. *Right,* normalized MST binding curves for different cMyBP-C fragments titrated against miniHMM. *D, left,* SDS-PAGE and structure of myosin S2Δ. *Right,* normalized MST binding curves for different cMyBP-C fragments titrated against myosin S2Δ. Means ± SEM, n = 3 to 6. cMyBP-C, cardiac isoform of myosin-binding protein C; MST, microscale thermophoresis; P/A, proline- and alanine-rich.

The regulatory N-terminal region of cMyBP-C spanning domains C0 to C2 (C0C2; Fig. 2*A*) has been shown to interact with cardiac myosin and was proposed to stabilize its OFF state both *in vitro* and *in situ* (8, 16, 17). Here, we determined the affinity and interaction specificity of the N-terminal region for the isolated myosin head and tail domains using MST.

C0C2 binds the isolated β-cardiac myosin head domain with a *K*_*d*_ of 3.4 ± 0.3 μmol/l (mean ± SEM, n = 4) (Fig. 2*B*, *black*; Table 1), which is about five times lower than the affinity of myosin S1 for the tail domain. Truncation of domain C2 and the m-motif (*i.e.*, the C0C1 fragment; Fig. 2*B*, *purple*) had no significant effect on the interaction with a similar *K*_*d*_ of ∼3 μmol/l, suggesting that the interaction between myosin S1 and NTD of cMyBP-C is mainly mediated by domain C0, the P/A linker or C1, or a combination thereof.

**Table 1:** Summary of equilibrium dissociation constants (*K*_*d*_ in μmol/l) for different cMyBP-C constructs binding to myosin S1, miniHMM, myosin S2Δ, and miniHMM-P as determined by MST

cMyBP-C fragment (domain composition)	Myosin S1	miniHMM	Myosin S2Δ	miniHMM-P
C0C2 (C0-P/A-C1-m-C2)	3.4 ± 0.3	3.4 ± 0.1	6.5 ± 0.6	2.7 ± 0.1
C0C2-3P (C0-P/A-C1-m-C2)	2.2 ± 0.2	9.7 ± 0.5	cnd	5.0 ± 0.1
C0C1 (C0-P/A-C1)	3.8 ± 0.4	10.3 ± 0.5	199.3 ± 14.3	—
C2C4 (C2–C3–C4)	0.38 ± 0.01	4.7 ± 0.3	292.4 ± 20.1	—
C5C7 (C5–C6–C7)	0.77 ± 0.1	3.2 ± 0.2	25.8 ± 0.8	—
C5C7-N755K (C5–C6–C7)	0.43 ± 0.04	—	73.9 ± 6.9	—
C5C7-R820Q (C5–C6–C7)	1.30 ± 0.04	—	59.1 ± 2.9	—
C5C7-R943Q (C5–C6–C7)	0.61 ± 0.05	—	32.6 ± 2.5	—
C0	91.3 ± 4.5	335.1 ± 17.7	cnd	—
C1	3.8 ± 0.2	17.0 ± 0.5	cnd	—
m-motif	183.1 ± 7.1	49.7 ± 3.8	41.4 ± 2.3	—
C2	42.3 ± 1.2	501.9 ± 57.6	cnd	—
C3	25.0 ± 0.6	—	—	—
C4	2.5 ± 0.1	—	—	—
cMyBP-C	0.30 ± 0.02	—	—	—
ΔC0C2-cMyBP-C	0.51 ± 0.02	—	—	—

Means ± SEM (n = 3–7).

Abbreviation: cnd, *K*_*d*_ cannot be reliably determined.

As a step toward further localizing the myosin S1-binding site, we determined its affinity for individual cMyBP-C domains. Domain C1 binds myosin S1 with a *K*_*d*_ comparable to that of the longer multidomain constructs (*K*_*d*_ = 3.8 ± 0.2 μmol/l, mean ± SEM, n = 3), suggesting that the main interaction site between the cMyBP-C NTDs and myosin S1 is localized to domain C1. In agreement, the isolated domains C0, C2, and m-motif showed only weak interaction with myosin S1 with *K*_*d*_ between 40 and 200 μmol/l, which is several orders of magnitude lower compared with the binding affinity of either C1 or myosin S2Δ.

In the intact myosin molecule, the regulatory light chains (RLCs) of the two myosin heads form close interactions with each other at the head–tail junction (Fig. 1*B*), which is believed to be important for the stabilization of the myosin OFF state, and can likely be regulated *via* phosphorylation of RLC (28). However, an intact RLC neck region is not present in the isolated myosin S1 used in the aforementioned experiments. We therefore produced a recombinant fragment of human cardiac myosin containing the myosin S2Δ tail domain bound to two RLCs, called “miniHMM” (29) (Fig. 2*C*). The C0C2 fragment binds miniHMM with a *K*_*d*_ of 3.4 ± 0.1 μmol/l (mean ± SEM, n = 3), comparable to the binding of C0C2 to myosin S1. A fragment lacking the m-motif and C2 (C0C1) retained binding to miniHMM, albeit with about three times lower affinity (10.3 ± 0.5 μmol/l; mean ± SEM, n = 3), indicating that either of these domains might contribute to the interaction. As before, we analyzed the contribution of each individual domain to miniHMM binding. Both domain C1 and the m-motif bind miniHMM with *K*_*d*_ of ∼15 and ∼50 μmol/l (Fig. 2*C* and Table 1), respectively, localizing the main interaction site to the C1/m-motif interface. Interestingly, the individual domains bind with weaker affinity to miniHMM compared with the larger C0C2 fragment, suggesting a multisite-binding mechanism.

The main interaction site of cMyBP-C with the myosin S2Δ tail was localized to the m-motif (Fig. 2*D* and Table 1), in very good agreement with previously published results (30). The isolated m-motif binds myosin S2Δ with a *K*_*d*_ of ∼40 μmol/l, in agreement with m-motif binding to miniHMM. Surprisingly, the affinity of C0C2 for myosin S2Δ (*K*_*d*_ of ∼7 μmol/l) is about sixfold higher compared with the m-motif alone, although none of the isolated Ig domains show significant binding to the myosin tail, suggesting that the m-motif structure is likely stabilized by the flanking domains. These results are in good agreement with cosedimentation binding assays of C0C2 with isolated cardiac myosin mini filaments (31).

### Phosphorylation of C0C2 abolishes interaction with myosin tails but not head domains

Phosphorylation of cMyBP-C in the cardiac-specific m-motif has been associated with a release of myosin head domains from the surface of the thick filament and an increase in myosin crossbridge cycling kinetics (23, 24, 32), suggesting that phosphorylation weakens the interactions between cardiac myosin S1, myosin S2, and cMyBP-C, and thereby favoring actomyosin crossbridge formation.

**Figure 3:**
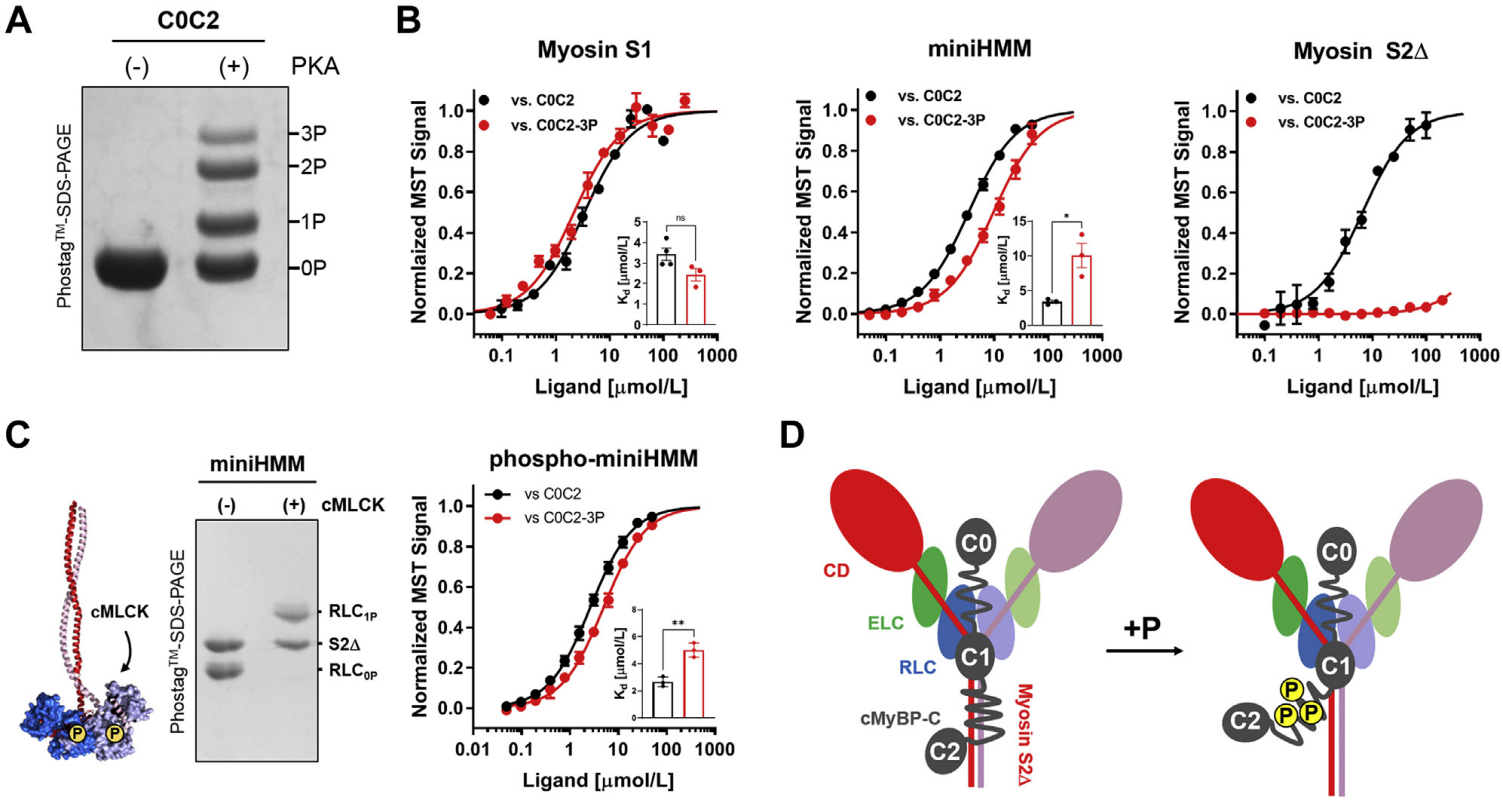
**Effect of phosphorylation on C0C2–cardiac myosin interactions.***A,* Phostag-SDS-PAGE of C0C2 before (−) and after *in vitro* phosphorylation with PKA (+). Individual phosphospecies are clearly separated by Phostag-SDS-PAGE and labeled accordingly. *B,* normalized MST binding curves for unphosphorylated (C0C2, *black*) and tris-phosphorylated C0C2 (C0C2-3P, *red*) titrated against cardiac myosin S1, miniHMM, and myosin S2Δ. *C, left, in vitro* phosphorylation of miniHMM by cMLCK resolved by Phostag-SDS-PAGE. *Right,* normalized MST binding curves for C0C2 (*black*) and C0C2-3P (*red*) titrated against phospho-miniHMM. *D,* cartoon representation for the effects of C0C2 phosphorylation on binding to myosin. See main text for details. Means ± SEM, n = 3 to 4. Statistical significance of differences between C0C2 and C0C2-3P was assessed with an unpaired two-tailed Student's *t* test: ∗∗*p* < 0.01, ∗*p* < 0.05, ns. CD, catalytic domain; cMLCK, cardiac isoform of myosin light chain kinase; C0C2, cMyBP-C spanning domains C0 to C2; ELC, essential light chain; ns, not significant; RLC, regulatory light chain.

To test this hypothesis, we *in vitro* phosphorylated recombinant C0C2 using PKA (Fig. 3*A*) and isolated the tris-phosphorylated species using ion-exchange chromatography (IEC) as described previously (17). Strikingly, tris-phosphorylation of C0C2 had no effect on its interaction with myosin S1 as indicated by almost identical *K*_*d*_ values (Fig. 3*B*, *left*; Table 1). This is consistent with the aforementioned observation that the m-motif has no or little contribution to the C0C2–myosin S1 interaction, which is mainly mediated by domain C1. Similarly, tris-phosphorylated C0C2 (C0C2-3P) retained its interaction with the miniHMM construct, albeit with reduced affinity by about a factor of 3 (*K*_*d*_ of 3.4 ± 0.1 μmol/l *versus* 9.7 ± 0.5 μmol/l for C0C2 and C0C2-3P, respectively; means ± SEM, n = 3) (Fig. 3*B*, *middle*). The decreased affinity of C0C2 for miniHMM after PKA phosphorylation is comparable to the effect of removing the m-motif from C0C2. This suggests that phosphorylation only abolished the interaction between the m-motif and the myosin S2Δ part of miniHMM but has no effect on the C1–miniHMM interaction. Consistent with this hypothesis, tris-phosphorylation of C0C2 completely abolishes the interaction with the isolated myosin tail domain (Fig. 3*B*, *right*).

However, phosphorylation of not only cMyBP-C but also RLC by the cardiac isoform of myosin light chain kinase (cMLCK) has been associated with an activating effect on the thick filament structure (33, 34) and an increase in force production, calcium sensitivity, and crossbridge kinetics of isolated cardiac muscle (35). Because of the similar structural and functional consequences, we hypothesized that RLC phosphorylation might affect the interaction of cardiac myosin with cMyBP-C.

We *in vitro* phosphorylated miniHMM using recombinant cMLCK to about 1 mol P_i_/mol RLC (Fig. 3*C*, *left*) and used the phospho-miniHMM construct in MST binding experiments. RLC phosphorylation had no effect on the interaction of unphosphorylated C0C2 with miniHMM (*K*_*d*_ of 3.4 ± 0.1 μmol/l *versus* 2.7 ± 0.1 μmol/l for miniHMM and phospho-miniHMM, respectively) (Fig. 3*C*, *right*). However, phosphorylation of miniHMM slightly increased its affinity for C0C2-3P as indicated by a decrease in the *K*_*d*_ from ∼10 μmol/l to ∼5 μmol/l (Fig. 3, *B* and *C*).

Taken together, these results suggest that cMyBP-C phosphorylation exclusively regulates the interaction of the m-motif with the myosin tails (Fig. 3*D*), and that RLC and cMyBP-C phosphorylation might independently regulate the myosin filament structure.

### High-affinity interaction sites of cMyBP-C with the myosin heads are localized to its central domains

In contrast to the N-terminal and C-terminal domains, the roles of the central region of cMyBP-C are not well understood. However, the majority of HCM-associated missense variants in the gene encoding for cMyBP-C have been localized to its central segment (21), suggesting that the native structure of those domains is necessary for normal cMyBP-C function.

**Figure 4:**
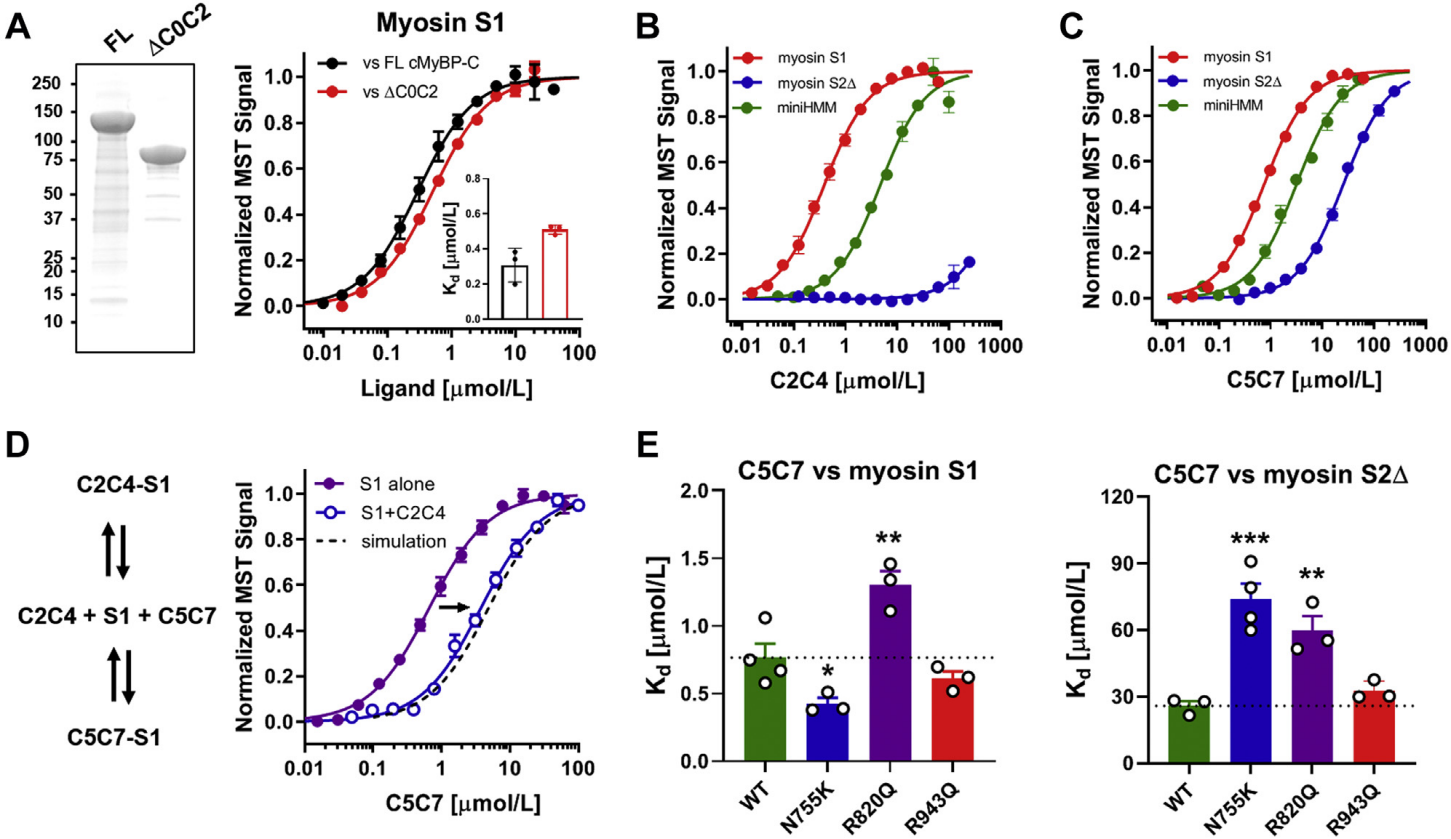
**The central domains of cMyBP-C tightly bind to the myosin heads.***A, left,* SDS-PAGE of purified full length (FL) and C0C2-truncated cMyBP-C (ΔC0C2). *Right,* normalized MST binding curves for full length (FL) (*black*) and ΔC0C2–cMyBP-C (*red*) titrated against myosin S1. *B,* normalized MST binding curves for C2C4 titrated against myosin S1 (*red*), myosin S2Δ (*blue*), and miniHMM (*green*). *C,* normalized MST binding curves for C5C7 titrated against myosin S1 (*red*), myosin S2Δ (*blue*) and miniHMM (*green*). *D, left,* competition model for C2C4 and C5C7 binding to the same site on myosin S1. *Right,* competitive titration of C5C7 into a mixture of myosin S1 and C2C4. The binding curves for C5C7 in the absence and presence of C2C4 are shown in *purple* and *blue*, respectively. The simulated data from the model are shown in the plot as a *black dashed line*. *E,* effect of the HCM-associated N755K (*blue*), R820Q (*purple*), and R943Q (*red*) substitutions in C5C7 on binding to myosin S1 (*left*) and myosin S2Δ (*right*). Means ± SEM, n = 3 to 4. Statistical significance of differences *versus* wildtype (WT) was assessed with a one-way ANOVA followed by Dunnett's multiple comparison: ∗*p* < 0.05, ∗∗*p* < 0.01, and ∗∗∗*p* < 0.001. cMyBP-C, cardiac isoform of myosin-binding protein C; C0C2, cMyBP-C spanning domains C0 to C2; HCM, hypertrophic cardiomyopathy; MST, microscale thermophoresis.

We hypothesized that the central domains of cMyBP-C are involved in its interaction with the myosin heads, and we tested this idea by comparing the binding of full-length and C0C2-truncated cMyBP-C (ΔC0C2-cMyBP-C) to isolated myosin S1 (Fig. 4*A*). cMyBP-C prepared from *Spodoptera frugiperda* insect cells (Sf9) has an endogenously low phosphorylation level (Fig. S1). Full-length cMyBP-C binds myosin S1 with a *K*_*d*_ of ∼0.3 μmol/l, similar to the affinity of myosin S1 for myosin S2Δ, but about an order magnitude higher than that of the isolated C0C2 fragment (Fig. 4*A*, *black*; Table 1). Moreover, ΔC0C2-cMyBP-C binds myosin S1 with roughly the same affinity (*K*_*d*_ of ∼0.5 μmol/l) (Fig. 4*A*, *red*). Taken together, these results suggest the presence of an additional high-affinity myosin head–binding site in either the central region or the C-terminal region of cMyBP-C. These values are in very good agreement with previously published data using human β-cardiac myosin and the C0C7 region of cMyBP-C lacking the C-terminal anchoring region (*K*_*d*_ of ∼1 μmol/l) (10), suggesting that the high-affinity myosin S1-binding site is localized within its central segment.

We analyzed the central domain architecture of cMyBP-C by comparing available high-resolution structures of individual domains and homology models of domains with unknown structure with its primary sequence (Fig. S2). The interdomain linkers between domains C2, C3, and C4 are very short and likely allow tight domain interface interactions, similar to those observed for tandem Ig domains of titin (36). In contrast, the m-motif preceding domain C2 and the longer linker connecting C4 and C5 are likely highly flexible and allow conformational plasticity, consistent with EM images of cMyBP-C showing kinks near both its N-terminal and central regions (37). Biochemical and biophysical characterization of both the individual domains and multidomain constructs suggest that domains in the C2C4 module are in close contact to each other *via* their loop regions and form a single structural unit (Fig. S3 and Supporting Information text). We tested the C2C4 module for binding to both myosin head and tail domains using MST (Fig. 4*B* and Table 1). C2C4 binds myosin S1 with a *K*_*d*_ of ∼0.4 μmol/l, similar to the binding affinity determined for full length and ΔC0C2-cMyBP-C but did not show any interaction with myosin tails. Moreover, C2C4 binds miniHMM with a *K*_*d*_ of ∼5 μmol/l, which corresponds to an about 10-fold lower affinity compared with myosin S1. The comparison suggests that the high-affinity interaction of myosin S1 with C2C4 requires either the essential light chain region or the myosin catalytic domain or both. Notably, the individual domains C2, C3, and C4 bind myosin S1 with a significantly lower affinity (*K*_*d*_ of 3–40 μmol/l; Fig. S4*A*), indicating that the myosin S1 interaction region is spread across the C2C4 module.

Similarly, domains C5 to C9 are connected *via* very short linkers, which could facilitate interdomain interactions and reduce the conformational flexibility of this cMyBP-C segment (Fig. S2). Domains C8 to C10 have been shown to tightly interact with both the myosin filament backbone and titin, and EM reconstructions of isolated thick filaments suggested a linear arrangement of these domains on the thick filament surface (4). We therefore tested the remaining domains C5 through C7 (C5C7) for binding to myosin head and tail domains using MST (Fig. 4*C* and Table 1). Notably, C5C7 binds myosin S1, miniHMM, and myosin S2Δ with *K*_*d*_ of ∼0.8, ∼3, and ∼25 μmol/l, respectively, suggesting that the central region of cMyBP-C exhibits *two* high-affinity binding regions (*i.e.*, in C2C4 *and* C5C7) for the myosin head domain. We further confirmed the interactions of C2C4 and C5C7 with the myosin head domains *via* cosedimentation experiments with artificial cardiac myosin filaments (Fig. S4*B*).

Two high-affinity binding regions in its central segment suggest that cMyBP-C might be able to simultaneously interact with the two myosin head domains of the same myosin molecule. In order to test this idea, we performed competition experiments and titrated increasing concentrations of C5C7 into a mixture of Alexa647-labeled myosin S1 and C2C4 (Fig. 4*D*). In the presence of C2C4, the EC_50_ for C5C7 binding to myosin S1 increased from ∼0.8 to ∼3.8 μmol/l, respectively, which is in very good agreement with a simple competition model (Fig. 4*C*, *dashed line*), suggesting that C2C4 and C5C7 compete for the same binding region on myosin S1. In contrast to C0C2, the *K*_*d*_ for both C2C4 and C5C7 binding to myosin S1 slightly increased at higher ionic strength, indicating that the interactions at least partially depend on electrostatic interactions (Fig. S5).

Moreover, we tested the binding of the central fragments to isolated bovine native thin filaments (NTFs) using a cosedimentation assay (Fig. S6, *A* and *B*). In contrast to the N-terminal fragment C0C2, which binds NTF in saturable manner with a *K*_*d*_ of ∼10 μmol/l (38, 39), the central regions C2C4 and C5C7 showed very little or no binding to NTFs, respectively. In very good agreement, both C2C4 and C5C7 had no effect on the NTF-stimulated myosin S1 ATPase activity, whereas C0C2 inhibited the NTF-stimulated myosin S1 ATPase by ∼50% in a concentration-dependent manner (Fig. S6*C*). These results show that the central segment of cMyBP-C (*i.e.*, C2C7) does not interact with the thin filament, which is in stark contrast to its strong binding to myosin S1.

HCM-associated nontruncating missense variants in cMyBP-C have been found to cluster in its central segment (21, 40), and we hypothesized that these mutations might affect the interaction of the central domains of cMyBP-Cs with cardiac myosin. To test this hypothesis, we generated C5C7 constructs carrying either the N755K, R820Q, or R943Q substitution in domains C5, C6, and C7, respectively. Homology modeling of domains suggests that all residues are surface accessible and likely do not affect protein folding, in good agreement with the soluble expression profile of these constructs in bacterial cultures (Fig. S7). Although both the N755K and R820Q substitutions only showed a small but significant effect on the interaction of C5C7 with myosin S1 (*K*_*d*_ of ∼0.7 μmol/l for wildtype *versus K*_*d*_ of ∼0.4 and ∼1.3 μmol/l for N755K and R820Q, respectively) (Fig. 4*E*, *left*), both variants strongly decreased the affinity for myosin S2Δ as indicated by *K*_*d*_ values about 2 to 3 times larger than those observed for the wildtype protein (Fig. 4*E*, *right*). In contrast, the R943Q variant did not show an effect on binding to either myosin S1 or myosin S2Δ. These results support the hypothesis that HCM-associated mutations in this region of cMyBP-C at least partially exert their pathological effect *via* altered interactions with cardiac myosin.

## Discussion

### The central domains of cMyBP-C tightly bind to the myosin motors

Although the structural effects of cMyBP-C binding to and activating the actin-containing thin filaments have been extensively studied (18, 19, 41, 42), the structural basis of the functional effect of cMyBP-C on the thick filament structure has remained largely elusive. Early studies suggested that the m-motif binds myosin S2 in a phosphorylation-sensitive manner (26), which would allow control of the myosin head orientation and therefore actomyosin-driven contractility *via* phosphorylation of cMyBP-C. More recent studies, however, have suggested a more complicated effect of NTD of cMyBP-C on the regulatory state of thick filament mediated by direct interactions with the force-generating myosin head domains (8, 10, 29).

Although our results agree with the hypothesis that NTD of cMyBP-C acts as a phosphorylation-sensitive regulatory element that binds to both the myosin head and tail domains, several independent lines of evidence suggest that the interaction sites with the highest affinity between cMyBP-C and myosin are *not* localized to its NTD (Fig. 4). First, the binding affinity of full-length cMyBP-C to myosin S1 is about an order of magnitude higher compared with the isolated C0C2 fragment. Second, removal of the C0C2 region from cMyBP-C had only a small effect on the binding affinity to myosin S1. Third, fragments of the central segment of cMyBP-Cs bind myosin S1 with about the same affinity as the full-length protein.

The present results therefore indicate that the interaction sites with the highest affinity between cMyBP-C and myosin S1 are localized to the central segment of cMyBP-C. Comparison of the primary sequence of the central segment of cMyBP-C with available high-resolution structures and homology models of individual domains shows a defined modular architecture with regions of low interdomain flexibility. Domains C2, C3, and C4 are connected *via* short three amino-acid linkers, which would allow close domain–domain interactions *via* their proximal loop regions. Biochemical and biophysical characterization of this region is in good agreement with a rigid arrangement of these domains, which is further supported by published small-angle X-ray scattering data on the C2C4 and C3C4 module, showing low conformational flexibility (43). A similar domain architecture was observed for the C5C7 module, although with some flexibility between C5 and C6 (43, 44).

Both C2C4 and C5C7 bind myosin S1 with about the same affinity as the full-length and C0C2-truncated cMyBP-C, indicating that the central segment of cMyBP-C has two independent high-affinity myosin S1-binding regions. Moreover, direct competition between C2C4 and C5C7 for myosin S1 binding indicates overlapping interaction sites on the myosin head domain as shown by MST (Fig. 4*C*). In the sarcomeric C-zone, three molecules of cMyBP-C are associated with three pairs of myosin heads out of the nine pairs in every 43 nm repeat (45). The stoichiometry and overlapping binding sites for myosin S1 suggests that a single cMyBP-C molecule can simultaneously interact with the two myosin heads of an individual myosin molecule. The central segment of cMyBP-C might therefore be directly involved in the stabilization of the myosin filament OFF state by crosslinking the so-called “blocked” and “free” head and stabilize the interacting-heads motif (IHM). Moreover, the C-terminal anchoring region (C8C10) of cMyBP-C has been associated with the free head of the proceeding myosin head pair (4), suggesting that cMyBP-C might also be able to crosslink myosin heads on adjacent crowns and facilitate intermolecular signaling between myosin molecules within the thick filament. The presence of multiple myosin-binding regions might also explain how cMyBP-C can control the conformation of the majority of myosin heads in the C-zone despite its low stoichiometry compared with myosin heads.

Taken together, these results suggest that the central region of cMyBP-C is not a passive scaffold that holds the NTDs in close proximity to the myosin head domains or actin as currently thought (46) but likely constitutes a dynamic regulatory element that contributes to the regulation of the activation state of myosin in the thick filament, the availability of myosin heads for contraction, and the associated ATP utilization. Notably, according to these results, cMyBP-C does not lie parallel to the thick filament axis in an extended conformation as previously thought, but its N-terminal and central domains are likely organized into a more compact docking platform. Although many structural details of these interactions remain unclear, their effect is to stabilize a population of myosin heads in a roughly helical lattice on the surface of the thick filaments, that is, the myosin OFF or IHM state observed in EM reconstructions of isolated thick filaments.

These results help to explain the etiology of heart failure associated with HCM-linked mutations in this region of cMyBP-C, which might interfere with myosin interaction and dysregulate the thick filament by increasing the number of myosin head domains in the functional ON state (Fig. 4*D*). Mutations might increase the energy demand of the heart and cause a hypercontractile phenotype—both hallmarks of HCM. In good agreement, nontruncating missense mutations appeared to cluster in the C3, C6, and C10 domains, which suggest that these domains are key regions for the native function of the protein (40).

### Potential role of the cardiac-specific C0 domain

The conserved structural features between the cardiac and skeletal muscle isoforms of MyBP-C suggest a similar mode of interaction between skeletal MyBP-C and skeletal muscle myosin. However, cardiac MyBP-C exhibits unique structural features, including an additional N-terminal Ig-like domain termed C0, with unknown functional significance.

The current experiments only show a weak interaction of C0 with cardiac myosin *in vitro*. However, the local or effective concentrations of both myosin and cMyBP-C were estimated to be in the high micromolar range, which would make a *K*_*d*_ of ∼90 μmol/l for myosin S1 binding functionally significant. A previous study suggested an interaction with the RLC region of myosin with *K*_*d*_ in the low micromolar range (29).

In contrast, C0 has been shown to readily interact with bare F-actin and isolated NTFs with low micromolar *K*_*d*_ in a position that does not interfere with tropomyosin's position on F-actin (19, 41). Moreover, binding of C0 to isolated NTFs has been proposed to enhance binding of domain C1, which competes for tropomyosin-binding sites on actin and can activate the thin filament structure in the absence of calcium.

Domains C0 and C1 are connected *via* a largely unstructured P/A-rich linker of 50 to 60 amino acid residues. The P/A linker is highly flexible and mobile in solution and can adopt both a folded and an extended conformation with a maximal extension of ∼8 nm (43). The maximally extended conformation would allow C0-PA/-C1 alone to bridge the interfilament distance with a maximal length of ∼18 nm, significantly longer than the estimated surface-to-surface distance of thick and thin filaments in the intact myofilament lattice (∼15 nm) (47).

**Figure 5:**
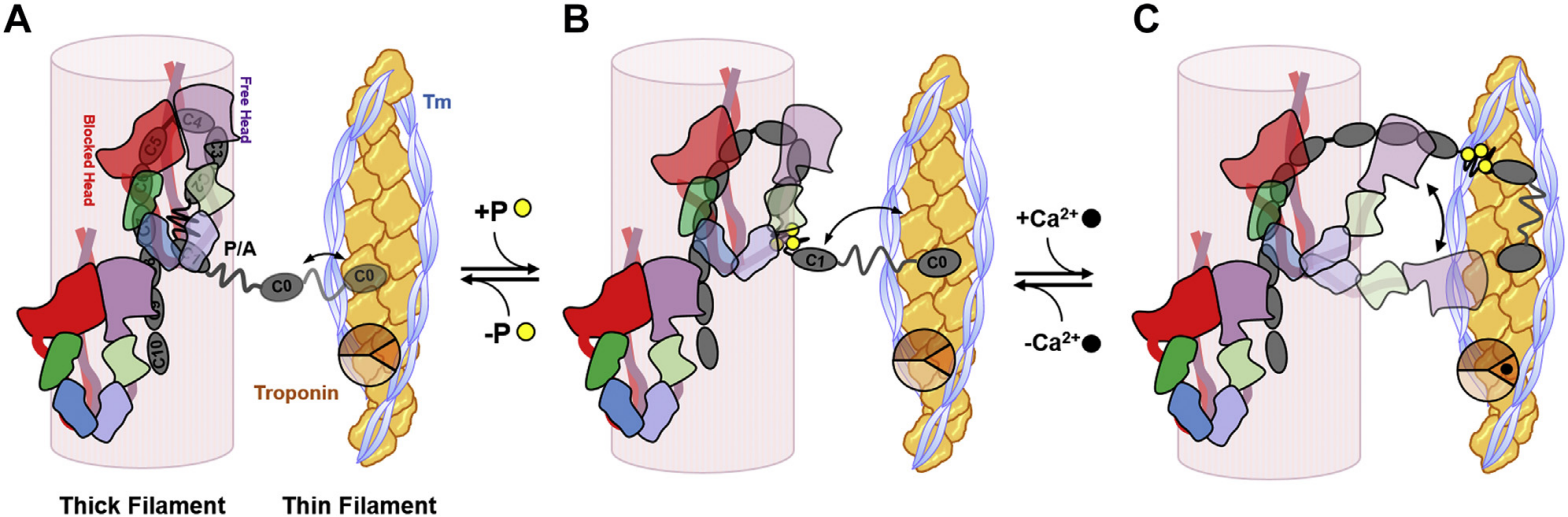
**Hypothetical model for the role of cMyBP-C during cardiac muscle activation.***A,* in the dephosphorylated state, cMyBP-C (*dark gray*) is bound to myosin catalytic domains of the myosin blocked head (*red*) and free head (*purple*) *via* interactions of its central domains (C2C4 and C5C7, respectively) and C1/m-motif with the RLC–neck region. The N-terminal domain C0 is in a conformational equilibrium between thin filament bound and unbound states, determined by the flexibility and elongation of the P/A linker. *B,* phosphorylation (*yellow circles*) of cMyBP-C abolishes the m-motif–myosin S2 interaction, which releases parts of cMyBP-C (and associated myosin heads) from the thick filament backbone, and increases the probability of C0–thin filament interactions. *C,* calcium activation of the thin filaments subsequently leads to cMyBP-C domain C1 cooperatively binding to available actin-binding sites in proximity to C0, which would fully activate the thin filament. In addition, this structural rearrangement would bring cMyBP-C–attached myosin heads closer to the surface of the calcium-activated thin filament and increase the probability of actomyosin crossbridge formation. cMyBP-C, cardiac isoform of myosin-binding protein C; P/A, proline and alanine-rich.

This suggests that although the domains C1 through C10 are bound to the surface of the thick filament *via* strong interactions with the filament backbone, and myosin head and tail domains, the NTD C0 can reach the actin-containing thin filament in the presence of the myosin filament OFF state (Fig. 5*A*). This might constitute a cardiac-specific interfilament signaling pathway that can coordinate the regulatory state of the thin and thick filaments during cardiac muscle activation.

Moreover, it was previously suggested that the P/A linker might act as a spring-like element, which shares high sequence homology to the proline-, glutamate-, valine-, and lysine-rich region of titin (48). The amino acid composition and length of the P/A linker correlates with the heart rate of mammals (49) and can be phosphorylated by physiologically relevant kinases such as glycogen synthase kinase 3-β (50), suggesting that the flexibility and length of the P/A linker and therefore thin filament binding of C0 can be tuned to a specific functional task of the heart muscle.

### Effects of cMyBP-C and myosin RLC phosphorylation

Although phosphorylation of cMyBP-C has important functional consequences for the myocardium, such as increased crossbridge kinetics and decreased myofilament calcium sensitivity, and is essential for the normal performance of the mammalian heart (51), phosphorylation of NTD of cMyBP-C had either no or small effects of its interaction with myosin. The only phosphorylation-sensitive interaction was observed between the cardiac-specific m-motif and myosin S2Δ. In contrast, C0C2 phosphorylation had no effect on the binding to myosin S1 and only weakened the interaction with miniHMM.

Moreover, the interaction of the central domains of cMyBP-C with myosin S1 are likely phosphorylation independent and might represent a constitutively bound state of the myosin motors, suggesting that phosphorylation does not abolish myosin–cMyBP-C interactions *per se* as currently thought (8, 26) but rather changes the conformation of the cMyBP-C–myosin head complex, which likely facilitates interaction of both the N-terminus of cMyBP-C and myosin heads with actin. For instance, the central segments C2C4 and C5C7 might compete with C0C2 for binding sites on miniHMM in a phosphorylation-sensitive manner, which might allow myosin heads to leave the folded OFF state upon phosphorylation of m-motif of cMyBP-C.

Similarly, RLC phosphorylation did not change the interaction of miniHMM with C0C2, supporting the idea that RLC and cMyBP-C phosphorylation independently regulate myofilament function. cMyBP-C and RLC are phosphorylated by different kinases operating downstream of distinct signaling pathways. Moreover, RLC phosphorylation is likely to affect the regulatory state of the myosin heads in the whole thick filament, whereas the effects of cMyBP-C phosphorylation are restricted to every third crown of myosin heads in the sarcomeric C-zone, although its regulatory effects can likely be communicated between myosin molecules *via* cooperative transitions in the thick filament structure implied by interaction between successive myosin heads in the thick filament OFF state (Fig. 4*E*) (4). In good agreement, RLC phosphorylation increases both the calcium sensitivity of isometric force and the rate of force redevelopment of rat ventricular trabeculae by roughly the same extent independent of the cMyBP-C phosphorylation background (28, 52, 53).

### Hypothetical model of cardiac myosin regulation by cMyBP-C

Our results are consistent with a hypothetical model in which cMyBP-C stays tightly associated with the myosin head domains during both the diastolic and systolic states of the myocardium mediated by the phosphorylation-sensitive interactions of the NTDs of cMyBP-C with the myosin neck/tail region and its central domains with the myosin heads.

The aforementioned results in combination with the studies cited in the introduction lead to a new working model for cardiac myosin regulation by cMyBP-C. In the diastolic OFF state, myosin heads are sequestered in the IHM *via* head–tail interactions, and myosin heads in the C-zone are further tethered to the surface of the thick filament *via* strong interactions of C-terminal domains (C8C10) of cMyBP-C with the filament backbone and its central region (C5C7 and C2C4) with the free head and blocked head of the succeeding myosin molecule, respectively. The OFF state is further stabilized *via* interactions of the m-motif and C1 domain with the myosin tail and head domains, respectively. In contrast, domain C0 is in a dynamic equilibrium between thin filament bound and unbound states, which is facilitated by the high flexibility and mobility of the P/A linker (Fig. 5*A*).

PKA phosphorylation of the m-motif during β-adrenergic stimulation abolishes its interaction with the myosin tail domain and weakens the interaction of NTD of cMyBP-C with the myosin neck region (Fig. 5*B*). Moreover, phosphorylation changes the structure of the NTD from an elongated to a more compact conformation and increases the mechanical stability of the m-motif (37, 54). This combined structural rearrangement brings both domains C0 and C1 closer to the surface of the thin filament (Fig. 5*B*). Since both domains have been shown to cooperatively bind to the thin filament, this would increase the probability of thin filament interactions of domain C1 (Fig. 5*B*, *double arrow*), which has been shown to have an activating effect on the thin filament structure by competing with tropomyosin for actin-binding sites (19). Because myosin heads strongly interact with the C2C4 segment, this structural rearrangement would bring the myosin catalytic domain of the free head closer to the surface of the thin filament.

Increase in the intracellular calcium concentration at the beginning of systole would subsequently trigger the activation of the remainder of the thin filament and allow NTDs of cMyBP-C and cMyBP-C-bound myosin motors to strongly attach to actin (Fig. 5*C*). Phosphorylation of m-motif of cMyBP-C would therefore favor actomyosin interactions by bringing myosin head domains tethered to the central segment of cMyBP-C closer to the surface of the thin filament, consistent with X-ray diffraction experiments on isolated cardiac muscle and electron micrographs of isolated thick filaments carrying constitutively phosphorylated cMyBP-C (24, 32). Conversely, at the end of systole, when the calcium transient has already reached diastolic levels, myosin heads associated with cMyBP-C would remain attached to actin because of cMyBP-C locally stabilizing the thin filament ON state (3, 16) and increasing the effective concentration of the myosin heads close to their actin-binding sites.

In this view of regulation, cMyBP-C directly controls the rate of myosin crossbridge attachment and detachment from actin *via* guiding myosin heads from the folded IHM OFF state into the actin-attached ON state and vice versa. The results described previously establish a new model for myosin-based regulation by cMyBP-C, its physiological control *via* phosphorylation, and impairment associated with heart disease and heart failure.

## Experimental procedures

### Protein production

Bovine β-cardiac myosin S1 was prepared from freshly frozen bovine heart ventricle as described previously (55). Alternatively, bovine β-cardiac myosin S1 was purchased from Cytoskeleton, Inc.

MiniHMM was expressed from a pET Duet-1 vector in BL21(DE3)-RIPL cells (Agilent Technologies; catalog no.: 230240) and purified by affinity chromatography on HisTrapFF columns (GE Healthcare). Histidine tag was removed by digestion with tobacco etch virus (TEV) protease, and the histidine tag and TEV protease removed by passing the digestion mixture through a 1 ml HisTrapFF column and collecting the flow through.

Myosin S2Δ, miniHMM, and cMyBP-C constructs were prepared as described previously (30). Briefly, proteins were expressed from a modified pET6a vector fused to an N-terminal histidine tag and TEV protease site in BL21(DE3)-RIPL cells. Following purification on HisTrapFF columns, the hisitidine tags were removed by treatment with TEV protease. Proteins were further purified by IEC on Mono S or Mono Q columns (GE Healthcare), concentrated to >100 μmol/l, snap-frozen in liquid nitrogen, and stored in aliquots at −80 °C for experiments. Purity was estimated by SDS-PAGE and electrospray ionization MS (ESI–MS) to >95%.

Full-length and C0C2-truncated cMyBP-C were expressed in *S. frugiperda* (Sf9) cells according to the manufacturer's instructions (Thermo Fisher Scientific) and purified by affinity chromatography on HisTrapFF columns followed by TEV digest of the N-terminal histidine tag and size-exclusion chromatography on Superose12 columns.

### Differential scanning fluorimetry

Proteins were mixed with 20× SYPRO Orange (Thermo Fisher Scientific) in PBS containing 1 mmol/l DTT, dispensed in a BioRad qPCR plate, and heated from 25 to 95 °C at a rate of 1 °C/min in a MX3005p qPCR machine (Agilent). Fluorescent emission at 610 nm following excitation at 492 nm was measured, with the resulting curve defined in Excel, and the *T*_m_ was calculated in GraphPad Prism (GraphPad Software, Inc).

### Fluorescence spectroscopy

Fluorescence emission spectra were recorded on FluoroMax Spectrofluorometer. Emission spectra were recorded with 1 nm excitation and emission filter slit and corrected for background fluorescence.

### Protein phosphorylation

Recombinant C0C2 was phosphorylated by incubation with the catalytic subunit of PKA (Calbiochem) and the tris-phosphorylated species was purified by IEC as described previously (17). MiniHMM was phosphorylated using the catalytic subunit of human cMLCK as described previously for isolated RLC (33). Phosphate incorporation and purity of phosphoproteins were estimated by Phostag-SDS-PAGE and ESI–MS.

### MST

MST experiments were performed on a Monolith NT.115 instrument (NanoTemper) in interaction buffer containing 20 mmol/l Mops, pH 7, 1 mmol/l MgCl_2_, 50 mmol/l KCl, 1 mmol/l DTT, and 0.05% (v/v) Tween-20. For experiments with the isolated m-motif, the pH was adjusted to 6.2.

Proteins were labeled with Alexa 647-NHS (Molecular Probes, Inc; Thermo Fisher Scientific) according to the manufacturer's instructions, and dye incorporation (efficiency of >80%) was confirmed by HPLC and ESI–MS. All proteins were either gel-filtered into and/or extensively dialyzed against interaction buffer. Titration experiments were performed with a fixed concentration of 100 nmol/l of Alexa647-labeled proteins in premium capillaries.

### NTF cosedimentation

NTFs were prepared according to previously published protocols, and cosedimentation experiments were performed as described (38).

### Myosin filament cosedimentation

Bovine cardiac myosin was prepared according to previously published protocols, and cosedimentation experiments were performed as described (31).

### Models of cMyBP-C domains and protein docking

Structures of cMyBP-C domains were generated in SWISS-MODEL (56) and energy minimized using ModRefiner (57).

### Statistical analysis

Data are represented as means ± SEM, with the number of experiments indicated by n. Statistical significance of the difference between two groups was assessed with an unpaired two-tailed Student's *t* test. Details of significance levels are shown in the figure legends.

## Data availability

The data supporting the findings of the study are available in the article and its Supporting Information. All remaining raw data will be available from the corresponding author upon reasonable request.

## Supporting information

This article contains supporting information.

## Conflict of interest

The authors declare that they have no conflicts of interest with the contents of this article.
