# Supplementary material for: Microscale thermophoresis suggests a new model of regulation of cardiac myosin function via interaction with cardiac myosin-binding protein C
Source: J Biol Chem. 2021 Dec 14;298(1):101485. doi: 10.1016/j.jbc.2021.101485 (PMC8733265; doi:10.1016/j.jbc.2021.101485)
Supplement: Supporting Information [file mmc1.pdf]

# Supporting Information

## Microscale thermophoresis suggests a new model of regulation of cardiac myosin function via interaction with cardiac myosin binding protein-C

Saraswathi Ponnam[1] and Thomas Kampourakis[1,*]

[1]Randall Centre for Cell and Molecular Biophysics; and British Heart Foundation Centre of Research Excellence, King's College London, London, SE1 1UL, United Kingdom

## Supporting Information Text

**Biophysical characterization of the C2C4 module.** Comparison of the high-resolution structures of domains C2, C3 and C4 with cMyBP-C's primary sequence suggests that these domains are in close contact to each other and potentially can form interactions at their domain interfaces (Fig. S2). All individual domains exhibit tryptophan fluorescence with an emission maximum between 325-330 nm, suggesting that the tryptophan residues are packed in the hydrophobic core of the Ig domains (Fig. S3A) and that the domains are folded. For unfolded domains tryptophan fluorescence is expected to occur at significantly longer wavelengths (>345 nm).

Next, we tested accessibility of the interdomain linkers in C2C4 using partial proteolytic digestion by trypsin (Fig. S3B). As a positive control, C0C2 was readily degraded by trypsin at already very low enzyme-to-substrate ratios, consistent with the idea that the P/A-linker and m-motif are highly flexible regions. In contrast, C2C3 is highly resistant to tryptic proteolysis, indicating that the interdomain linker is not solvent accessible and C2C3 forms a rigid arrangement with tight interdomain interactions. Similarly, C3C4 is resistant to proteolytic digestion by trypsin, especially compared to the C0C2 control. However, some degradation was observed at higher enzyme-to-substrate ratios.

Next, we analyzed the unfolding behavior of individual domains and multi-domain constructs using Differential Scanning Fluorimetry (DSF) (Fig. S3C). C1mC2 shows two transitions at ~43°C and ~63°C, suggesting that domains C1 and C2 unfold independently in the multidomain construct. In contrast, C2C3, C3C4 and C2C4 show a single transition in the thermal denaturation profile with melting temperatures ($T_m$) of 58.1°C, 49.5°C and 48.8°C, respectively. Moreover, the $T_m$ of the multidomain constructs correspond well with the $T_m$ of the domain with the lowest temperature stability. Taken together, this suggests a cooperative unfolding behavior, consistent with strong interdomain interactions within the C2C4 module.

**Supporting Information Figures and Figure Legends**

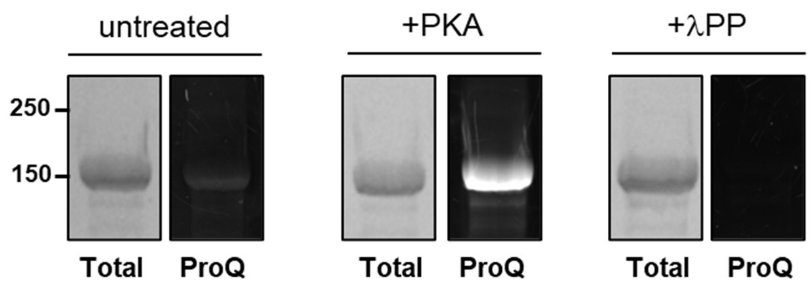


**Figure S1.** Phosphorylation level of cMyBP-C purified from *Spodoptera frugiperda 9 (Sf9)* cells. SDS-PAGE of purified untreated (left), PKA phosphorylated (middle) and λ-protein phosphatase (λPP) treated (right) cMyBP-C. Gels were stained with either Coomassie (total protein stain) or Pro-Q Diamond phospho-protein stain.

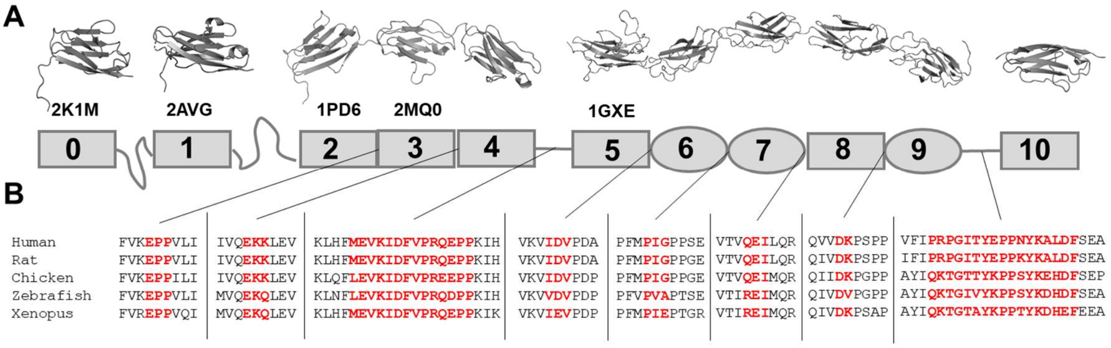


**Figure S2**. Analysis of cMyBP-C domain and linker structures. (A) Structure of individual cMyBP-C domains obtained from either the PDB archive (PDB codes are indicated under the domains) or by homology modelling using SWISS-MODEL (https://swissmodel.expasy.org, please see main text for reference). (B) Multiple species sequence alignment of cMyBP-C interdomain linkers. The linker sequences are highlighted in red.

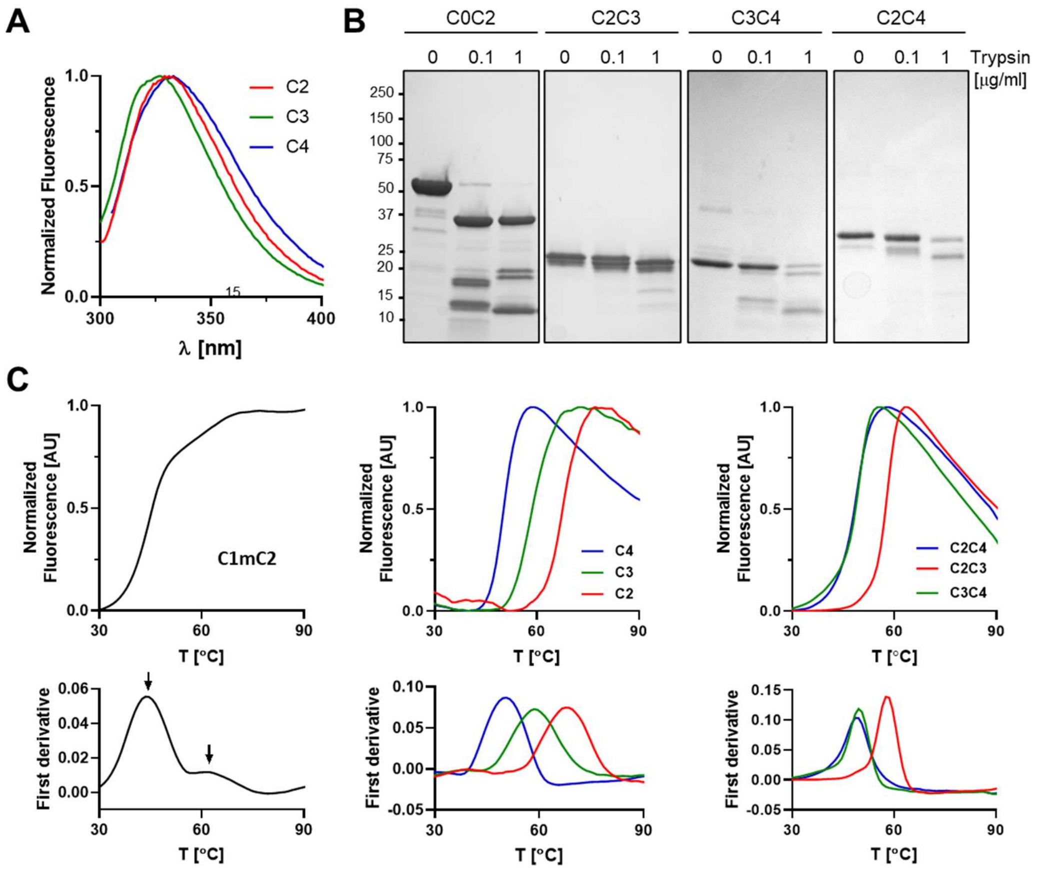


**Figure S3.** Biophysical and biochemical characterization of the C2C4 module. (A) Tryptophan fluorescence of domains C2 (red), C3 (green) and C4 (blue). (B) Partial tryptic proteolytic digestion of cMyBP-C multidomain constructs at different protease concentrations. Please note that the C0C2 control is readily degraded by trypsin. (C) Differential scanning fluorimetry (DSF) of C1mC2 (left), and individual domains and multi-domain constructs of the C2C4 module.

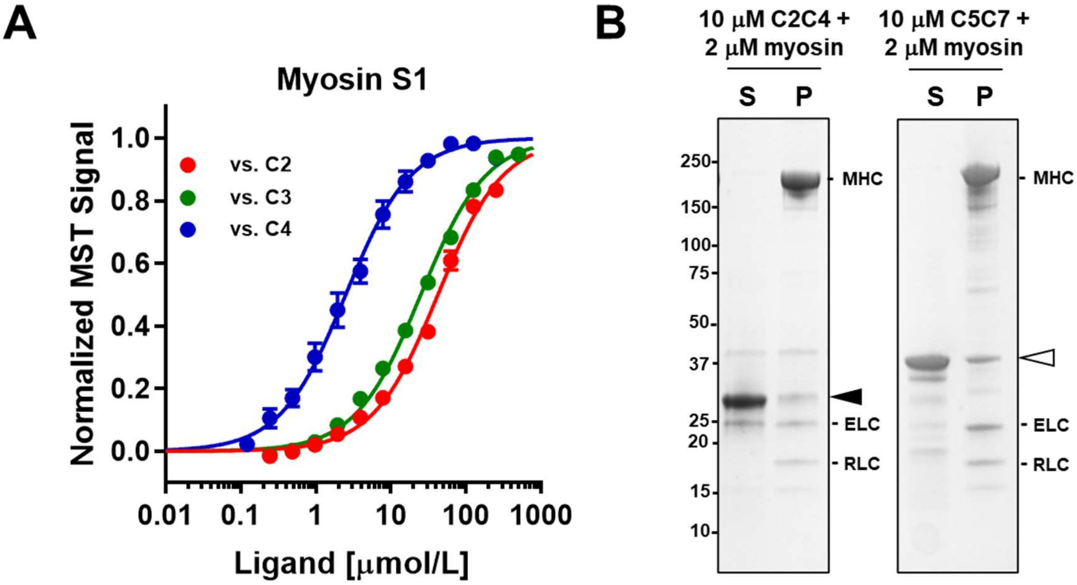


**Figure S4.** (A) Normalized MST binding curves for Alexa647-labelled myosin S1 titrated against increasing concentrations of C2 (red), C3 (green) and C4 (blue). Means ± SEM, n=3. (B) Myosin co-sedimentation assay with C2C4 and C5C7. Please note that both C2C4 (black arrowhead) and C5C7 (white arrowhead) co-sediment with myosin filaments (S-soluble fraction; P-pellet fraction).

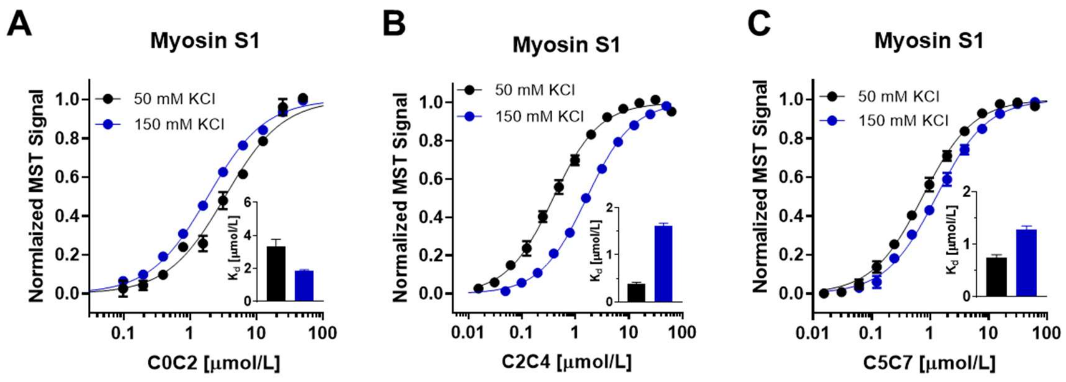


**Figure S5.** Effect of ionic strength on protein-protein interactions determined by Microscale Thermophoresis. Normalized MST binding curves for (A) C0C2, (B) C2C4 and (C) C5C7 titrated against myosin S1 in the presence of 50 mmol/L (black) and 150 mmol/L KCl (blue). Means ± SEM, n=2-4.

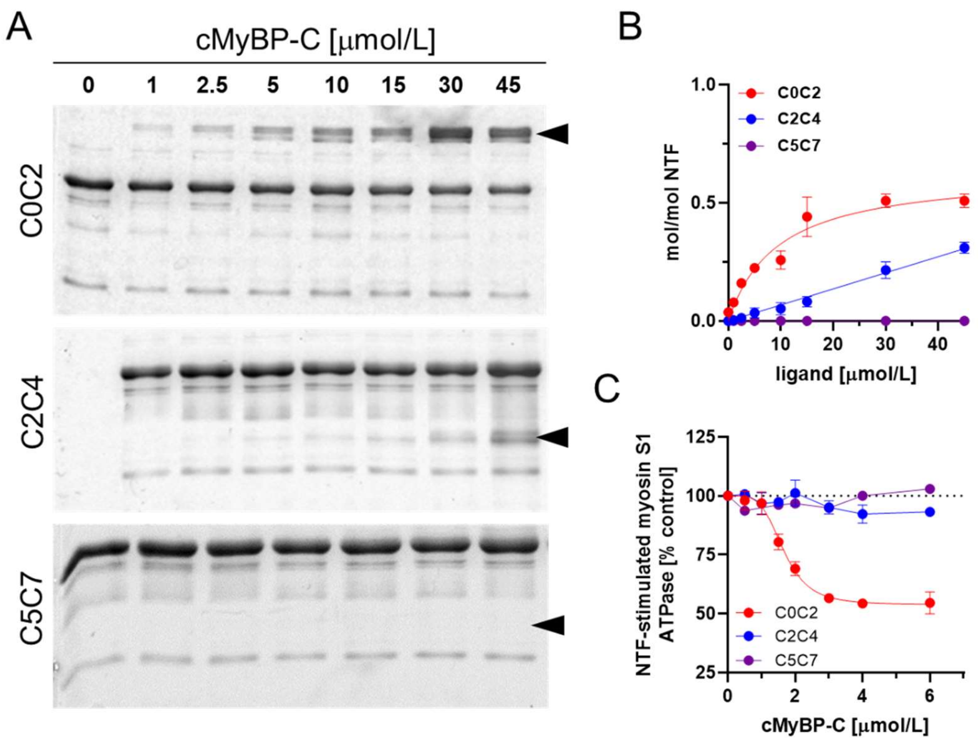


**Figure S6.** Binding of cMyBP-C fragments to bovine native thin filaments. (A) SDS-PAGE of co-sedimentation of native thin filaments (NTF) with C0C2 (top), C2C4 (middle) and C5C7 (bottom). The position of the cMyBP-C fragments is indicated by arrowheads. (B) Normalized binding curves for C0C2, C2C4 and C5C7 to NTF. (C) NTF-stimulated myosin S1 ATPase activity measurements in the presence of increasing concentrations of C0C2, C2C4 and C5C7 at pCa 4 (100 μmol/L free $Ca^{2+}$). C0C2 decreased the ATPase activity in a concentration-dependent manner.

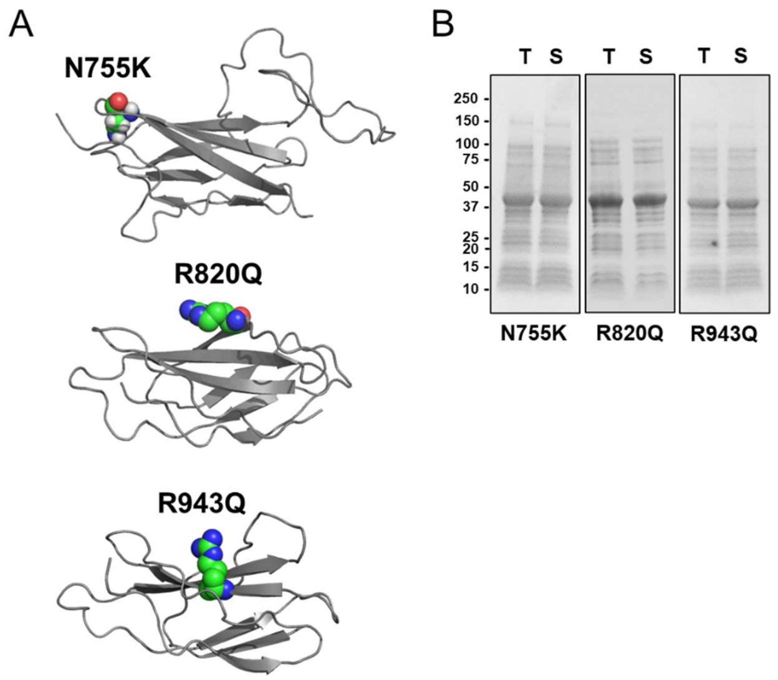


**Figure S7.** (A) Structure of human cMyBP-C domain C5 (PDB 1GXE; top), and homology models of domains C6 (middle) and C7 (bottom) with HCM-associated mutations (N755K, R820Q and R943Q) shown in van-der-Waals representation. (B) Expression profile analysis of bacterial cultures expressing variant containing C5C7 constructs. Cultures were lysed, separated into total (T) and soluble fractions (S) and run on SDS-PAGE.
